# Mesenchymal Stem Cells Regulate the Innate and Adaptive Immune Responses Dampening Arthritis Progression

**DOI:** 10.1155/2016/3162743

**Published:** 2016-10-26

**Authors:** R. A. Contreras, F. E. Figueroa, F. Djouad, P. Luz-Crawford

**Affiliations:** ^1^Laboratorio de Inmunología Celular y Molecular, Centro de Investigación Biomédica, Facultad de Medicina, Universidad de los Andes, Santiago, Chile; ^2^Programa Doctorado en Biomedicina, Facultad de Medicina, Universidad de los Andes, Santiago, Chile; ^3^Inserm, U 1183, 34091 Montpellier, France; ^4^Université de Montpellier, 34000 Montpellier, France

## Abstract

Mesenchymal stem cells (MSCs) are multipotent stem cells that are able to immunomodulate cells from both the innate and the adaptive immune systems promoting an anti-inflammatory environment. During the last decade, MSCs have been intensively studied* in vitro* and* in vivo* in experimental animal model of autoimmune and inflammatory disorders. Based on these studies, MSCs are currently widely used for the treatment of autoimmune diseases such as rheumatoid arthritis (RA) characterized by complex deregulation of the immune systems. However, the therapeutic properties of MSCs in arthritis are still controverted. These controversies might be due to the diversity of MSC sources and isolation protocols used, the time, the route and dose of MSC administration, the variety of the mechanisms involved in the MSCs suppressive effects, and the complexity of arthritis pathogenesis. In this review, we discuss the role of the interactions between MSCs and the different immune cells associated with arthritis pathogenesis and the possible means described in the literature that could enhance MSCs therapeutic potential counteracting arthritis development and progression.

## 1. Introduction

Rheumatoid arthritis (RA) is a chronic autoimmune disease that mainly affects large and small joints resulting in bone and cartilage erosion but can spread to multiple body systems, including cardiovascular, pulmonary, and skeletal systems [1, 2]. The etiology of RA is not completely understood but the main pathophysiological process triggering RA is an abnormal activation of T cells, B cells, natural killer (NK) cells, dendritic cells (DCs), macrophages, and neutrophils which produce proinflammatory mediators such as cytokines, proteinases, and growth factors mediating joint destruction and systemic complications [3, 4]. Currently, there is no treatment for RA or strategies to manage symptoms and reduce the progression of the disease [5]. Thus, novel strategies aiming at both reducing inflammation and inducing tissue regeneration in order to improve RA progression are currently investigated [5].

Mesenchymal stem cells (MSCs) have been reported as a potential candidate for the treatment of RA due to their regenerative and anti-inflammatory properties that could both induce the regeneration of damaged joints and modulate the pathogenic immune responses [6].

## 2. Mesenchymal Stem Cells

MSCs are self-renewal multipotent stromal cells that are able to differentiate into cells of the mesenchymal lineage [7]. The International Society for Cellular Therapy (ISCT) has established the minimal criteria for identifying MSCs. These criteria include a fibroblastic-like morphology, the expression of markers such as CD90, CD105, and CD73, the lack of expression of hematopoietic markers such as CD45, CD34, and CD14, and the capacity to differentiate into adipocytes, chondrocytes, and osteocytes [8]. They have been successfully isolated from almost all postnatal and mesodermal tissues including bone marrow, placenta, adipose tissue, dental tissue, and menstrual blood [9–12]. They can be cultured easily* in vitro *through plastic adherence under regular culture conditions [8]. The subsequent* in vitro* culture generates a heterogeneous population of stromal cells able to secrete several factors and cytokines including vascular endothelial growth factor (VEGF), fibroblast growth factor (bFGF), insulin growth factor (IGF), and hepatocytes growth factor (HGF). These trophic factors produced by MSCs have been described to promote angiogenesis and inhibit apoptosis and fibrotic remodeling while inducing cell proliferation [13, 14].

Another function displayed by MSCs is their capacity to modulate both the innate and the adaptive immune responses. Indeed, MSCs inhibit the activation of dendritic cells (DCs), proinflammatory M1-like macrophages, natural killer (NK) cells, and B and T cells while inducing the generation of cells with anti-inflammatory phenotypes [15–18]. Based on these broad immunomodulatory abilities, the therapeutic potential of MSCs in autoimmune and inflammatory disorders has been intensively investigated in experimental mouse models [19–23]. Thus, as an experimental model of arthritis, the model of collagen induced arthritis (CIA) has been used with promising results [23–25]. However, according to the tissue sources and the strain of mouse used to isolate MSCs, discrepancies in their immunosuppressive properties and therapeutic potential have been reported [26–28]. This might be due to intrinsic molecular particularities of MSCs isolated from different sources or the impact of the microenvironment* in vivo*. Therefore, in order to safely and efficiently use MSCs for the long-term treatment of patients with RA, it is critical to understand the molecular mechanisms mediating MSCs immunosuppressive properties, the role of the pathological microenvironment which faces administrated MSCs on their function, and the impact of the dialogue between MSCs and the immune cells on their phenotypes.

## 3. Innate Immunity in RA and the Effect of MSCs

In RA, joints are infiltrated with various inflammatory cell types from both the innate and adaptive immune systems which interact to trigger joint destruction [4]. Macrophages are among the main players of RA [29]. They are described as highly plastic cells able to rapidly respond to a variety of stimuli adopting thus different phenotypes among which proinflammatory (M1) or anti-inflammatory (M2-like) macrophages are the two extremes subtypes [30, 31]. Initially, macrophages were described as a rare cell population within synovial fluids of patients and therefore were thought to be not significant for the RA diagnostic [32]. Later on, in the synovial membranes of patients with RA, the presence of phagocytic proinflammatory HLA-DR^+^ macrophages was observed [33]. These cells were mainly present in the synovial lining, and their numbers were also significantly increased in the adjacent tissue. This observation gave rise to the hypothesis that macrophages in the synovial membranes of patients with RA might initiate T cell infiltration and activation via antigen presentation triggering B cell infiltration and creating a positive inflammatory feedback [33].

Several studies have shown that TNF-*α* and IL-1*β*, mainly secreted by M1 macrophages, are abundant in RA, while IL-10, characteristic of M2-like macrophages, is lower in patients with RA as compared to healthy individuals [34]. In line with this study, it was shown that IL-10 knock-out mice develop exacerbated CIA and that the phenotype of synovial macrophages in these animals corresponds mainly to proinflammatory M1-like macrophages [35]. Moreover, in response to TNF-*α* and IL-1*β*, activated synovial fibroblasts produce high levels of receptor activator of nuclear factor-*κ*B (NF-*κ*B) ligand (RANKL) and macrophage colony-stimulating factor 1 (M-CSF) which are essential for formation of osteoclasts by fusion of myeloid precursors of monocytes/macrophages [36–38]. In healthy individuals, there is a balance between bone reabsorption and formation, governed by osteoclasts and osteoblasts, respectively, while in RA osteoclast activity is chronically induced, causing severe bone destructions [39, 40]. Macrophages also contribute to disease progression by producing reactive oxygen species (ROS), nitric oxide intermediates, and matrix-degrading enzymes as well as CXCL8 (also known as IL-8) and monocyte chemoattractant protein-1 (MCP-1, also known as CCL2) which are crucial for neutrophil and monocyte recruitment in the joint [2].

As previously mentioned, MSCs have shown promising results for RA treatment. In this context, it has been described that adipose-derived MSCs (ASC) significantly reduced clinical symptoms of arthritis in a CIA mouse model [23]. This effect has been associated with their capacity to inhibit RANK-induced osteoclastogenesis, leading to bone loss in the presence or absence of proinflammatory cytokines, in both contact-dependent and independent manner [41].

In addition to their capacity to prevent osteoclast formation, MSCs also participate in the regulation of the phenotypic switch from proinflammatory M1-like to IL-10 producing M2-like macrophage subsets [42]. The mechanism by which MSCs modulate macrophage polarization in RA has not been fully elucidated yet. However, under inflammatory conditions, it has been demonstrated that MSCs can increase the secretion of PGE2 through the upregulation of COX2 expression as well as other components of the arachidonic acid pathway, presumably promoting the polarization of macrophages toward an M2-like phenotype [43]. Moreover, upon treatment with TNF-*α*, MSCs secrete high levels of TNF-*α*-stimulated gene 6 protein (TSG-6), an anti-inflammatory molecule. TSG-6 will then prevent the interaction of the Toll-like receptor 2 (TLR-2) on macrophages by interacting with their CD44 receptor and thus modulate TLR-2-mediated NF-*κ*B signaling and decrease macrophage inflammatory response [44]. We and others have shown that IL-1 receptor antagonist (IL-1RA) produced by MSCs is involved in the modulation of the plasticity of macrophages promoting their differentiation toward an M2 phenotype [18, 45].

Dendritic cells (DCs), potent stimulators of adaptive immunity, play a critical role in the establishment and maintenance of immunological tolerance. In RA, DCs are the main inducer of inflammation by presenting antigens to autoreactive T cells that in turn produce different cytokines associated with T helper cells differentiation [46]. Since DCs have the capacities to modulate T cells response, a promising new immunotherapeutic strategy for the treatment of RA is through the generation of tolerogenic DCs (tDCs) which play an important role in inducing peripheral tolerance by promoting regulatory T (Treg) cells and suppressing effector T cells [47]. Indeed, synthetic PPAR-*γ* agonists such as rosiglitazone, a selective ligand for PPAR-*γ*, induce the generation of tolerogenic DC- (tDC-) like anti-inflammatory function which improves CIA progression [48].

MSCs are able to interfere with the maturation of DCs, generating tDCs, by impairing the TLR activation both* in vitro* and* in vivo *[15, 49, 50].* In vitro,* upon exposure to MSCs, the expressions of some activation surface markers on DCs are downregulated and are no longer able to process and present antigen to T cells, resulting in significantly decreased T cell proliferation [49]. The production of cytokine by LPS-activated DCs cocultured with MSCs was inhibited by paracrine mediators acting on the mitogen-activated protein kinase (MAPK) cascade of the NF-*κ*B pathway, which is upregulated upon TLR-4-induced DCs activation. In line with this study, an impairment of antigen-specific naive T cell priming* in vivo* after an intravenous administration of MSCs was reported. This was associated with a significant decrease of the cells number in the draining lymph nodes resulting from a decreased migration capacity of DCs which could be, in part, explained by a decreased expression of CCR7 and CD49d*β*1 involved in the homing of DCs to lymphoid organs [51]. IL-6, highly produced by MSCs, was described as one of the main mediators of MSCs immunoregulatory effect on DCs [52]. Natural killer (NK) cells have an important role in the defense against microbial agents and tumor cells. Additionally, NK cells can affect the adaptive immunity by producing cytokines and killing directly other immune cells, which indicate a regulatory role of NK cells in autoimmunity [53]. In RA, NK cells have been reported for both their protective and detrimental roles in arthritis progression. NK cells are abundant in the joints of RA patients and express RANKL and M-CSF. They are normally associated with CD14^+^ monocytes triggering their differentiation into osteoclasts in the synovial membrane [54]. The depletion of NK cells from mice before the induction of CIA reduces the severity of arthritis and significantly prevents bone erosion, suggesting the pivotal role of NK cells in the destruction of bone observed in RA patients [54]. However, upon NK cell depletion, immunized mice displayed an early onset of arthritis with more severe clinical symptoms, which correlated with increased B cell generation, autoantibody production, and a marked increase in the number of IL-17-secreting cells in the synovial tissue [55, 56]. All together, these results suggest that NK cells may play also a protective role in the development of experimental arthritis, an effect that might be mediated by suppressing Th17 cells.

MSCs and NK cells have been shown to interact* in vitro*. The outcome of this interaction may depend on the NK cell activation state and/or on the cytokines present in the milieu. Thus, the cytokine-induced proliferation of freshly isolated, resting NK cells is highly susceptible to MSC-mediated inhibition. Moreover, the function of NK cell is regulated by several receptors that can generate either inhibitory or activating signals. Exposure of resting NK cells to activating cytokines, such as IL-2, increases the expression of the activating receptors NKp44, CD69, NKp30, and NKG2D [57]. MSCs can significantly inhibit IL-2-induced NK cells proliferation and also prevent the induction of their effector functions, such as cytotoxic activity and cytokine production, mostly driven by indoleamine 2,3-dioxygenase (IDO) and prostaglandin E2 (PGE2) as crucial mediators of the MSCs immunosuppressive effect on NK cells [16]. Moreover, it has been reported that human NK cells secrete NAP-2 (CXCL7), a chemokine that can induce MSC migration. The use of specific antagonists of CXCR2, a receptor that recognizes NAP-2, abolished NK cell-mediated MSC recruitment [58]. However, when NK cells are activated, they can also recognize allogeneic MSCs and induce the apoptosis of the latter cells [59]. This could be reversed by activating MSCs with IFN-*γ* through high levels of HLA, B-C expression [60]. In the context of arthritis, the specific effect of MSCs on NK cells has not been determined yet.

## 4. Adaptive Immunity in RA and the Effect of MSCs

As previously mentioned, in RA pathogenesis, innate immune cells have an important role not only by directly inducing inflammation and bone erosion but also by recruiting and activating different cells from the adaptive immunity including T and B lymphocytes. A key event in the pathogenesis of RA associated with the adaptive immunity is the production of autoantibodies [4]. Most of the antibody reactivity described occurs before the onset of disease and in most individuals the autoantibodies stock is developed already at the onset of disease. Only very few individuals appear to be autoantibody-positive later during the disease course [61]. Consequently, B cell depletion therapy with anti-CD20 antibody (rituximab) has become an important biologic therapy with positive clinical results [62]. Indeed, B cell depletion reduces rheumatoid factor and anti-citrullinated protein antibodies (ACPA), which are prevalent biomarkers of RA [62]. Therefore, autoreactive B cells participating in antigen presentation, costimulation, and cytokine production likely play an important role but are not the main mediators of RA, since a decrease in autoantibodies does not necessarily correlate with clinical outcome [63]. In this context, it has been shown that the T cell population is altered after B cell depletion, resulting in reduced T cell activation and cytokine production. In proteoglycan-induced arthritis (PGIA), an experimental murine model of arthritis, Treg cell percentage was elevated in B cell-depleted mice, compared to control treated mice that exhibited a higher proportion of CD4^+^ T cells expressing Foxp3 and CD25 [62]. Of note, CD4^+^CD25^+^ cells from B cell-depleted mice expressed higher amounts of Foxp3 and were significantly more suppressive than those from the control group. Interestingly, when Treg cells were removed with an anti-CD25 monoclonal antibody simultaneously with B cell depletion therapy, the severity of PGIA was decreased to the level of untreated mice [62]. Thus, B cells have the capacity to regulate the inflammatory responses in arthritis, in part, by educating T cells within a regulatory phenotype. However,* in vitro* studies on the role of MSCs on B cells are controversial. Indeed, while the inhibitory properties of MSCs on B cells proliferation and differentiation to plasma cells as well as antibody production have been described, the capacities to induce the survival and stimulate proliferation and differentiation of various subsets of purified B cells derived from both healthy donors and systemic lupus erythematosus (SLE) patients have been shown [64]. These controversies might be explained by the* in vitro *culture conditions, the origin of the cells used by the different laboratories, and the complex interaction between MSCs, B cells, and T cells. Indeed, T cell signaling is required for MSCs to exert their immunomodulatory effect on B cells, which appears to be dependent on soluble factors that are released when the three cell types are in direct contact [65]. In line with this hypothesis, the interaction between programmed cell death 1 (PD-1) protein and its ligand (PD-L1) expressed by IFN-*γ*-activated MSCs and B cells was shown to be required for MSC-mediated inhibition of B lymphocytes activation [66]. Blocking PD-1 or PD-L1 restores the molecular pathways associated with B cell stimulation and partially rescues B cell proliferation [66]. Assessing the role of soluble factors, Asari et al. demonstrated that MSCs are able to inhibit the mRNA expression levels of the B lymphocyte-induced maturation protein-1 (Blimp-1), a master transcriptional regulator required for B cell terminal differentiation in a cell contact independent manner [67]. Furthermore, conditioned media derived from MSCs inhibit B cells differentiation* in vitro* and* in vivo*. When applied to mice immunized with both T cell-independent and T cell-dependent antigens, MSCs significantly suppress the antigen-specific immunoglobulin M and G1 secretion [67]. The mechanism by which MSCs modulate B cells differentiation and proliferation is still under investigation. The role of the chemokine CCL2 on the immunomodulatory capacity of MSCs on B cells was suggested since MSCs isolated from lupus-like mice and SLE patients have an impaired inhibition activity on B cells proliferation and differentiation. In line with this hypothesis, it has been reported that the expression of the CCL2 on MSCs derived from SLE patients or lupus-like mice is lower than that in healthy or wild-type MSCs and that CCL2 overexpression in MSCs derived from SLE patients restored their immunosuppressive function on B cells [68]. Recently, VEGF secretion by MSCs has been shown to have an important role in the survival of B cells through the activation of the AKT signaling pathway that inhibits the expression of caspase-3 [69]. MSCs directly promote the development of CD19^+^CD24^high^CD38^high^ IL-10-secreting regulatory B cells through the chemokine stromal derived factor-1*α* (SDF-1*α*) and its receptor, CXCR7, which contribute to the generation of an immunosuppressive environment [70, 71]. Finally, in arthritis, we have shown that MSCs inhibit plasmoblasts generation* in vivo* in CIA through the expression of the IL-1 receptor antagonist (IL-1RA) on MSCs [18].

As previously mentioned, T cells play a key role in the pathogenesis of RA. Already in 1975, it was observed that T cells were present in the synovial membrane of patients with RA [72]. Later on, both CD4^+^ and CD8^+^ T cell subsets were identified in the joints of RA patients [33, 73]. CD8^+^ T cells are cytotoxic cells that induce cell death of virus-infected and cancer cells via the release of cytolytic granules or the induction of Fas-mediated apoptosis [74]. In RA, the pathogenic role of CD8^+^ T cells is not well described; however, a high association between HLA class I polymorphisms and a higher probability to develop RA has been observed, as well as a correlation between the number of CD8^+^ T cells in the joint and the severity of the disease [75]. This study supports the hypothesis that CD8^+^ T cells, found at a high frequency in the inflammatory joints, could play a role in RA pathogenesis [76]. MSCs are able to suppress CD8^+^ activation* in vitro *[77]. Moreover, it has been recently described that MSCs are able to induce the generation of CD8^+^CD28^−^ Treg cells and also enhance their ability to suppress CD4^+^ T cell proliferation and activation [78]. The generation of these latter regulatory T cells subsets after MSCs injection in the context of arthritis has not been investigated yet.

CD4^+^ T cells, also known as T helper (Th) cells, exert multiple roles in the control of the immune response including B cells differentiation and function and CD8 activation [79]. CD4^+^ T cells can be polarized into different T helper subsets depending on the type of immune response required by the organism. Currently, the most studied CD4^+^ cells subtypes include T helper type 1 (Th1), T helper type 2 (Th2), T helper type 17 (Th17), and T regulatory (Treg) cells [80]. The different T helper subpopulations can be distinguished by their specific cytokine profile (IFN-*γ* for Th1, IL-4 for Th2, and IL-17 for Th17), their specific transcription factors (T-bet for Th1, GATA3 for Th2, ROR*δ*t and ROR*α* for Th17, and Foxp3 for Treg) [79, 81], and the combinations of chemokine receptors such as CCR6, CCR4, and CD161 for Th17, CXCR3 and CCR5 for Th1, and CCR4 for Th2 [79, 82].

Initially, Th1 cells were considered as the main T cell subset involved in RA. Indeed, early studies reported the presence of IFN-*γ*-secreting CD4^+^ T cells in the synovium of patients with RA [83, 84]. When IL-12, a Th1 polarizing cytokine, was neutralized using an anti-IL-12p40 antibody (one of the two subunits of IL-12 in CIA mice), the severity of the disease was attenuated [85]. However, when CIA was induced on IL-12p40 knock-out mice, 20% of mice still developed arthritis [85]. Moreover, the specific genetic ablation of the IL-12p35 subunit (the other subunits that compose IL-12) exacerbated arthritis symptoms [86]. Later on this was better understood with the discovery of the IL-23 cytokine that shares the IL-12p40 domain with IL-12. Interestingly, while IL-23, which is not secreted by Th1, does not influence Th1 differentiation process, it participates in the generation and maintenance of the proinflammatory Th17 lineage [87]. When IFN-*γ* or its signaling pathway was inhibited, the onset, development, progression, and severity of arthritis were increased in parallel with an increase of IL-17 levels in the serum and joints of CIA mice [88]. These results strongly suggest that Th17 cells are key effectors in arthritis [89].

The use of soluble IL-17 receptor-Fc on CIA mice to block IL-17 improved the disease progression in a dose-dependent manner. Conversely, the overexpression of IL-17 in the knee joint of type CIA mice accelerated the onset of the disease and aggravated the synovial inflammation confirming the critical role of Th17 in joint destruction [90]. CD4^+^ T cells display high plasticity. Th17 cells share differentiation pathways as well as molecular signatures with the anti-inflammatory Treg cells [91]. Treg cells are characterized by the high expression of CD25 and the transcription factor Foxp3, which is essential for Treg cell function [92]. Treg cells control inflammation using numerous suppressive mechanisms including both soluble and membrane-bound factors [93]. Moreover, Foxp3 deficiency in both human and mouse is responsible for the development of various autoimmune diseases [94]. Th17 and Treg cells plasticity might have been acquired initially to enable a flexible immune response for dealing rapidly with pathogens and to avoid an exacerbate inflammatory response. However, this cell plasticity can also lead to deregulation of immune responses and subsequently to the development of autoimmune diseases [95]. Thus, in RA, the increased frequency of Th17 cells has been proposed to be due to either a reduction in the number of Treg cells or a qualitative defect in their function [96]. The immunosuppressive properties of MSCs were first described in a mix lymphocytes reaction, where the capacity of MSCs to inhibit T cells proliferation was demonstrated [97]. Later on, it was described that MSCs are able to inhibit proinflammatory Th1 and Th17 cells and to induce Treg cells* in vitro* and* in vivo* [17, 98, 99]. Indeed,* in vitro*, MSCs inhibit the differentiation of naive CD4^+^ T cells into Th17 cells as well their capacity to secrete IL-17, IL-22, and TNF-*α*. The suppressive effect on memory Th17 cells is associated with an increased expression of Foxp3 and production of the anti-inflammatory cytokine IL-10 and is mediated by a cell to cell contact that depends on the PD-1 pathway [100, 101]. The inhibitory properties of MSCs on Th17 cells occur through cell-cell contact involving CCR6 and CD11a/CD18 expressed by T cells and their respective ligands, CCL20 and CD54, present on primed MSCs cultured with inflammatory cytokines. The suppressive effect of MSCs on Th17 cells is also mediated by paracrine mechanisms including the production of PGE2 by MSCs which will bind to its receptor, EP4, on T cells and TGF*β*1 [100, 102]. In line with this study, the capacity of MSCs to induce CD4^+^CD25^high^Foxp3^+^ regulatory T cells from CD4^+^ T cells differentiating into Th1 and Th17 cells has been shown [17]. Similarly, MSCs direct the conversion of Th17 cells into Treg cells through an IL-17A^+^FoxP3^+^ double-positive phenotype [103] and the generation of a Th1 producing IL-10 cells [104]. The capacity of MSCs to inhibit proinflammatory Th1 and Th17 cells and to induce Treg cells has been demonstrated using several experimental mouse models. Indeed, administration of umbilical cord derived MSC (UC-MSC) in the sepsis model reduces the progression of the disease by inducing a population of CD4^+^CD25^+^Foxp3^+^ classical Treg cells in the lymph nodes of treated animals compared to the nontreated mice [105]. In arthritis, the beneficial effect of gingival derived MSC is associated with an increased frequency of CD4^+^CD39^+^Foxp3^+^ Treg cells and an inhibition on Th1 and Th17 lineages [24]. In accordance with this study, we have recently demonstrated that the therapeutic potential of murine bone marrow MSC in arthritis is associated with the generation of IL-10-producing regulatory Th17 cells in the draining lymph nodes of MSC-treated mice [106]. Of note, MSCs do not constitutively express suppressive factors such as PGE2 or PD-L1; rather they need to be bathed in proinflammatory milieu to adopt a regulatory phenotype enabling them to modify the immune response by modulating the cytokine secretion profile of T cell subsets in favor of a regulatory phenotype.

## 5. MSCs Preconditioning to Improve Their Therapeutic Features for Arthritis Treatment

As mentioned before, MSCs priming with IFN-*γ* or the stimulation of TLR signaling is required to induce their immunosuppressive phenotype [107, 108]. While IFN-*γ* normally acts as an enhancer signal for T cell activation and expansion, when used for the pretreatment of MSC, this cytokine drives the production of different mediators, some of them specific for human MSCs such as indoleamine 2,3-dioxygenase (IDO) or murine MSCs such as nitric oxide (NO) and other factors shared among species including PD-L1, ICAM, PGE2, and IL-6. All together, these factors produced by licensed MSCs will generate a T cell immunosuppressive environment [25, 109–111]. Several other proinflammatory cytokines such as TNF-*α*, IL-1*α*, or IL-1*β* have been described to further enhance the effect of IFN-*γ* on MSC priming [110]. MSC stimulation with IFN-*γ* combined with TNF-*α* augments the secretion of IL-8, IL-6, hepatocyte growth factor (HGF), and prostaglandin E2 production [112]. Furthermore, MSC priming with IFN-*γ* plus TNF-*α* has been shown to activate superoxide dismutase 3, an antioxidant and anti-inflammatory enzyme that catalyzes the dismutation of two superoxide radicals into hydrogen peroxide and oxygen [113]. IFN-*γ* and TNF-*α* combination has also been demonstrated to induce the production of chemokines such as CCR5, CCR10, CXCL9, CXCL10, and CXCR3, which are involved in the chemotaxis and the inhibition of immune effector cells [114]. This priming of MSC has been shown to inhibit CD4^+^ and CD8^+^ T cells, B cells, and NK cells [115].

**Figure 1:**
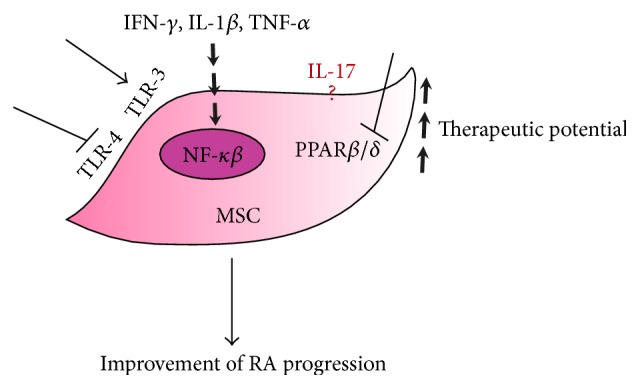
Preconditioning of MSCs to improve their therapeutic potential for arthritis treatment. Proinflammatory cytokines such as IFN-*γ*, IL-1*β*, and TNF-*α* and activation of the TLR-3 pathways are able to increase the immunosuppressive potential of MSCs probably through the activation of NF-*κβ* activity. PPAR*β*/*δ* inhibition, either chemical or genetic, also increased the therapeutic potential of MSCs. TLR-4 inhibition could also enhance the immunosuppressive potential of MSCs. The specific role of IL-17 in the suppressive effect of MSCs still remains a subject of controversy.

Thus, MSCs are sensitive to their microenvironment, making them able to sense and identify exogenous and endogenous danger signals, through the expression of different Toll-like receptors (TLR). Indeed, the expressions of TLR-1, TLR-2, TLR-3, TLR-4, TLR-5, and TLR-6 have been reported in human and mice MSCs [116]. Expression and function of TLR can be modulated in different ways in MSC. For example, hypoxia can significantly increase the mRNA expression levels of TLR-1, TLR-2, TLR-5, TLR-9, and TLR-10 [117]. Remarkably, the inflammatory environment may also modulate the pattern and function of TLR expressed by MSC. When cultured in the presence of IFN-*α*, IFN-*γ*, TNF-*α*, and IL-1*β*, the expression level of TLR-2, TLR-3, and TLR-4 was increased, while TLR-6 was downregulated [118]. The use of specific TLR ligands has shown that the triggering of different TLR resulted in the secretion of cytokines and chemokines on MSC. Depending on the TLR ligand encountered, MSC can polarize toward proinflammatory or immunoregulatory phenotypes. Indeed, following the activation of TLR-3 or TLR-4 with their respective agonists poly(I:C) or LPS, MSCs will exhibit different phenotypes: a TLR3-primed phenotype expressing immunosuppressive factors and the TLR4-primed MSC with a proinflammatory signature [119] (Figure 1).

More recently, it was shown that IL-17 together with IFN-*γ* and TNF-*α* can enhance the immunosuppressive effect of MSC. IL-17 was found to modulate the mRNA stability of ARE/poly(U)-binding/degradation factor 1 (AUF1), which is abundant in lymphoid organs and regulates the mRNA expression of various immune-related molecules including iNOS and IL-6. The role of IL-17 on AUF1 was further confirmed using AUF1^−/−^ MSC, which, after incubation with IFN-*γ* and TNF-*α*, further enhanced MSC immunosuppressive function, both* in vitro* and* in vivo*, without the addition of IL-17 [120]. Conversely, using olfactory ecto-MSCs (OE-MSCs), which are a population of stem cells that reside in the olfactory lamina propria, IL-17 was shown to significantly decrease the suppressive capacity of OE-MSCs on CD4^+^ T cells by downregulating the levels of inhibitory factors produced by OE-MSCs such as NO, IL-10, TGF-*β*, and PD-L1. Notably, IL-17 treatment inhibited the capacity of OE-MSCs in generating Treg cells as well as their capacity to suppress the generation of Th1 and Th17* in vitro* and* in vivo* using the CIA model. Furthermore, knockdown of IL-17R in OE-MSCs significantly enhanced their therapeutic effect in reducing CIA progression [121]. Although the studies compared MSCs from different sources known to display some discrepancies in their functions [26], further investigations are required to better understand the effect of IL-17 on MSC immunomodulatory capacity (Figure 1).

Finally, we have recently demonstrated that the modulation of the activity of peroxisome proliferator-activated receptor (PPAR) *β*/*δ* on MSCs improves their immunomodulatory effects. PPAR*β*/*δ* exhibits multiple biological functions including anti-inflammatory activities through the inhibition of NF-*κ*B signaling and cell adhesion molecule expression [122]. PPAR*β*/*δ* inhibition in MSCs significantly increased the immunosuppressive capacities of MSCs* in vitro* through the activation of NF-*κβ* signaling, resulting in the induction of the expression of iNOS and adhesive molecules. Moreover, using the CIA model, we showed that the inhibition of PPAR*β*/*δ* in MSCs significantly improved their therapeutic potential reducing the progression of arthritis. Therefore, these results not only place PPAR*β*/*δ* as a master regulator of the immunosuppressive properties of MSCs but also propose a novel strategy to enhance MSCs therapeutic potential (Figure 1) [123].

## 6. Conclusion 

The immunoregulatory abilities of MSCs have been studied for several years, demonstrating the wide repertory of mechanisms used by the cells to interact with the multiple actors of immune responses. Thus, in the context of arthritis involving macrophages, DCs, NK cells, T cells, B cells, and other cell types, MSCs appeared as candidate of choice for RA treatment. However, although the immunosuppressive effects of MSCs have been shown to alter the functions of several immune cells individually, the phenotypic plasticity of MSCs governed by their environment might alter MSC therapeutic effect. Importantly, the stable priming of MSCs toward an immunoregulatory state should be considered in order to optimize MSCs-based therapy.
